# Single-Cell Sequencing: Genomic and Transcriptomic Approaches in Cancer Cell Biology

**DOI:** 10.3390/ijms26052074

**Published:** 2025-02-27

**Authors:** Ana Ortega-Batista, Yanelys Jaén-Alvarado, Dilan Moreno-Labrador, Natasha Gómez, Gabriela García, Erika N. Guerrero

**Affiliations:** 1 Faculty of Science and Technology, Technological University of Panama, Ave Justo Arosemena, Entre Calle 35 y 36, Corregimiento de Calidonia, Panama City, Panama; ana.ortega6@utp.ac.pa (A.O.-B.);; 2 Gorgas Memorial Institute for Health Studies, Ave Justo Arosemena, Entre Calle 35 y 36, Corregimiento de Calidonia, Panama City, Panama; 3 Sistema Nacional de Investigación, Secretaria Nacional de Ciencia y Tecnología, Edificio 205, Ciudad del Saber, Panama City, Panama

**Keywords:** single-cell sequencing, tumor microenvironment, tumor heterogeneity, cancer research, precision medicine, artificial intelligence

## Abstract

This article reviews the impact of single-cell sequencing (SCS) on cancer biology research. SCS has revolutionized our understanding of cancer and tumor heterogeneity, clonal evolution, and the complex interplay between cancer cells and tumor microenvironment. SCS provides high-resolution profiling of individual cells in genomic, transcriptomic, and epigenomic landscapes, facilitating the detection of rare mutations, the characterization of cellular diversity, and the integration of molecular data with phenotypic traits. The integration of SCS with multi-omics has provided a multidimensional view of cellular states and regulatory mechanisms in cancer, uncovering novel regulatory mechanisms and therapeutic targets. Advances in computational tools, artificial intelligence (AI), and machine learning have been crucial in interpreting the vast amounts of data generated, leading to the identification of new biomarkers and the development of predictive models for patient stratification. Furthermore, there have been emerging technologies such as spatial transcriptomics and in situ sequencing, which promise to further enhance our understanding of tumor microenvironment organization and cellular interactions. As SCS and its related technologies continue to advance, they are expected to drive significant advances in personalized cancer diagnostics, prognosis, and therapy, ultimately improving patient outcomes in the era of precision oncology.

## 1. Introduction

Human cancers are complex ecosystems composed of cells with distinct phenotypes, genotypes, and epigenetic states. Current cancer study models do not adequately reflect tumor composition in patients [1]. Single-cell sequencing has revolutionized our understanding of cellular heterogeneity within cancer tissues, unlike traditional bulk RNA-seq analysis, which provides an average transcriptional profile of a tissue sample, masking cellular diversity [2]. Single-cell RNA sequencing (scRNA-seq) enables the dissection of complex tumor ecosystems at single-cell resolution, revealing rare cell types, transition states, and intercellular interactions vital for cancer progression and therapeutic response [3,4,5].

Single-cell sequencing (SCS) has revolutionized cancer research by providing a powerful platform to investigate clonal evolution, intratumoral heterogeneity, and treatment resistance [1]. This technology enables the analysis of genomic and transcriptomic profiles at the individual cell level for the identification of specific cellular subpopulations within tumors associated with disease progression and treatment response [6].

The advent of scWGS has transformed our understanding of cancer biology by providing an unprecedented view of the complex genetic landscapes of tumors at a single-cell level [7]. This technique involves isolating individual cells, amplifying their DNA, and then sequencing the genomes to understand genetic variations at a single-cell level [6]. It has also revealed the remarkable heterogeneity present both within and between cancer cells [8]. Meticulously analyzing the genomic profiles of single cells uncovered the trajectories driving tumor progression, the mechanisms underlying cancer cell metastasis, and the development of resistance to medical treatments [8,9].

This review examines how single-cell technologies have changed cancer biology research. It explores the technology’s key contributions to understanding critical aspects of cancer, including tumor heterogeneity, evolution, and the tumor microenvironment through its ability to analyze genomic, transcriptomic, and epigenomic profiles at the individual cell level. This review covers the essential capabilities of single-cell technology, its integration with other single-cell omics approaches, and the crucial role of advanced computational methods in data analysis. Additionally, it discusses the emergence of complementary technologies like spatial transcriptomics and in situ sequencing, while also addressing the potential future impact of these advancements on personalized cancer medicine. Throughout, this review emphasizes how these technological developments are driving progress in cancer diagnostics, prognostics, and therapeutic strategies, ultimately aiming to improve outcomes for cancer patients through more precise and personalized approaches.

## 2. Overview of Core Chemistry and Methods of Single-Cell Sequencing

To perform any single-cell sequencing assay, an isolation of individual cells is required from the heterogeneous system of interest before sequencing. One of the most widely utilized methods to purify thousands of single cells is FACS (fluorescence-activated cell sorting) [10] and MACS (magnetic-activated cell sorting) [11]. Advancements in isolation techniques have been driven by microfluidic technologies, enabling high-throughput cell processing. An example is droplet-based systems, such as the 10x Genomics Chromium platform, which encapsulates individual cells within nanoliter-sized water droplets containing barcoded beads. These beads are embedded with oligonucleotides carrying unique barcode sequences, allowing the transcripts from each cell to be accurately traced back to their cell of origin. Although hundreds of single cells can be processed simultaneously using this technology, strict quality control is needed to prevent numerous artifacts [12]. Microwell-based isolation is an alternate strategy in which cells are isolated in separate wells for further processing. Although it has a lower throughput than droplet-based systems, this technique is ideal for research that expects full-length transcript sequencing [13]. The type of isolation and preparation used is crucial since it affects data accuracy, cell recovery rates, and sequencing results. FACS and well-based techniques offer greater sensitivity for uncommon or delicate cell types, while high-throughput droplet-based techniques are best for profiling huge populations [14].

**Figure 1:**
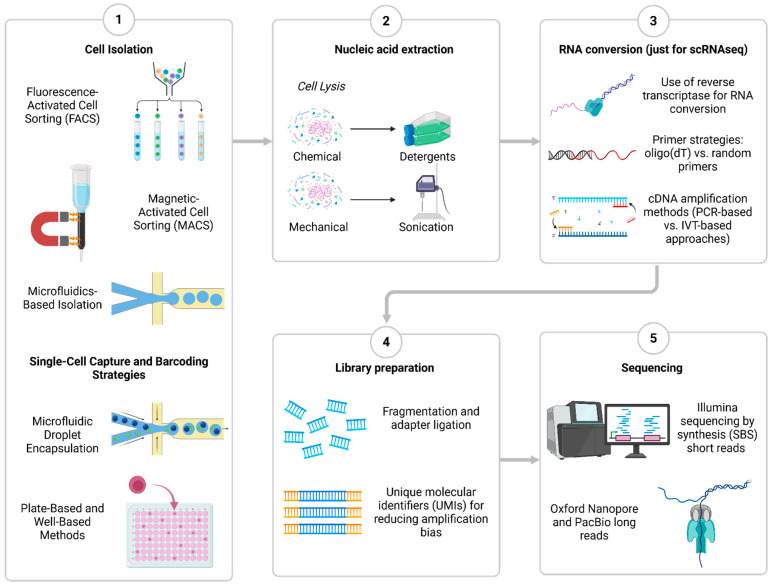
Core chemistry and methods of sc sequencing. 1. Most common cell isolation, capture, and barcoding strategies. 2. Nucleic acid extraction methods. 3. RNA conversion for transcriptomic assays. 4. Library preparation overview. 5. Sequencing using short or long reads. Created in www.BioRender.com. Accessed on 25 February 2025.

After isolation, the next step is cell lysis for nucleic acid extraction. However, preserving RNA integrity during lysis is a significant challenge, and quality control to verify DNA quality or RNA integrity should be evaluated. If performing scRNASeq, the extracted RNA must be converted into complementary DNA (cDNA). This process is typically mediated by reverse transcriptase enzymes, which use either oligo (dT) primers that target polyadenylated mRNA or random hexamer primers that capture a broader range of transcripts and amplification steps required since single-cell RNA is present in minute amounts. Two primary methods are used for cDNA amplification: polymerase chain reaction (PCR)-based approaches, such as Smart-seq2, and in vitro transcription (IVT)-based strategies, such as those used in the CEL-seq method [15]. Then, library preparation is performed to make the samples compatible with NGS platforms. Besides the usual DNA fragmentation and sequencing adapter addition, it is crucial to incorporate unique molecular identifiers (UMIs) which are short barcode sequences that help reduce amplification bias and allow accurate quantification of transcripts [13]. Finally, the step before data acquisition is typically performed using Illumina’s sequencing-by-synthesis (SBS) method with short read lengths. Alternatively, scSeq could be performed in long-read platforms such as Oxford Nanopore and PacBio [14]. The choice of sequencing technology depends on the research objectives, as short-read sequencing is more accurate for gene expression quantification, regulatory element analysis, and general transcriptomics; thus, long-read sequencing provides a more comprehensive view for alternative splicing analysis, isoform diversity characterization, and structural variant detection [16] (Figure 1).

## 3. Cancer Research

Within the conceptual framework of SGS, three central capabilities stand out: fidelity, co-presence, and phenotypic association. These capabilities are essential for understanding the unique advantages of scWGS. The fidelity capability refers to its ability to detect DNA features such as mutations or modifications existing at low levels of mosaicism within a sample. Unlike bulk DNA sequencing [17], the co-presence capability in single-cell DNA sequencing refers to its ability to capture multiple genomic features within a single cell [17]. This capability allows for the simultaneous analysis of various aspects of the genome, including copy number variations (CNVs), single-nucleotide variants (SNVs), structural variations such as insertions and deletions, chromosomal rearrangements, and DNA methylation patterns. This provides a more comprehensive understanding of the genetic landscape of individual cells. Furthermore, the phenotypic association capability enables the linking of genomic information with cellular phenotypes [17,18]. By connecting genetic data with specific cellular characteristics, researchers can gain insights into the functional implications of genomic variations at the single-cell level [17,18,19].

The co-presence and phenotypic association capabilities are particularly valuable in heterogeneous samples, spanning applications from profiling cells within an organism to distinguishing between tumor and normal cells or analyzing cellular mixtures in complex environments such as soil samples or the gut microbiome [17]. One of the fundamental applications of scWGS in cancer research is the characterization of circulating tumor cells (CTCs). CTCs are tumor cells that have detached from the primary tumor and entered the circulatory or lymphatic systems [20,21,22,23]. Understanding their role in metastasis may contribute to improved therapeutic management [24]. In a study conducted by Polzer et al., the scWGS technique was used to analyze the genomic profiles of CTCs in breast cancer patients, enabling the identification of fundamental principles guiding the evolution of individual tumors. These principles include the ability of tumor cells to generate genetic diversity through mutations and chromosomal rearrangements, as well as the clonal selection of subpopulations with evolutionary advantages, such as therapy resistance or increased metastatic capacity. These findings offer a new perspective on CTC dynamics, highlighting the coexistence of genetically distinct subpopulations, each with unique metastatic potentials and therapeutic vulnerabilities [25].

In addition to its application in studying CTCs, scWGS has enabled researchers to unravel the complex clonal architecture of solid tumors. This has revealed the hierarchical organization of genetically distinct subclones within a single tumor mass [26]. This approach has unveiled rare subpopulations of cells such as cancer stem cells or therapy-resistant clones, which can drive disease progression and treatment failure [26,27,28]. By reconstructing the evolutionary trajectories of these subclones, scWGS has provided valuable insights into the mechanisms underlying tumor heterogeneity and the emergence of resistant phenotypes [29].

By comparing single-cell data across multiple patients, researchers can identify common mutational patterns and chromosomal mutations that may contribute to the development and progression of specific cancer types [30,31]. This knowledge can inform the development of targeted therapies and guide personalized treatment strategies tailored to each patient’s unique genetic profile [31,32,33].

**Figure 2:**
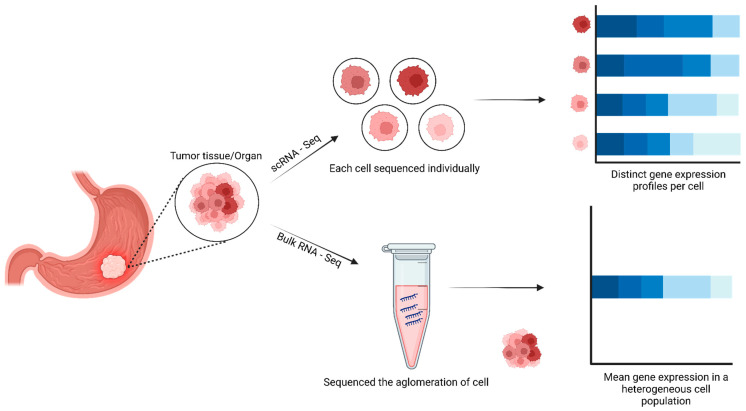
Workflow for scRNA-seq and bulk RNA sequencing. scRNA-seq reveals the distinct transcriptome of individual cells (top), while bulk RNA sequencing captures the average gene expression profile across all cells combined (bottom). Created in www.BioRender.com. Accessed on 23 October 2024.

Furthermore, single-cell sequencing has allowed the exploration of the intricate interaction between cancer cells and their microenvironment. By analyzing the genomic and transcriptomic profiles of individual cancer cells within the tumor environment, researchers can elucidate the complex dynamics between cancer cells, immune cells, and stromal components (Figure 2) [26]. These recent developments have improved the understanding of complex interactions between tumors and their immune microenvironment in various human cancers, which would allow the development of effective immune therapies [34,35].

As the field of single-cell technologies advances, the integration of multi-omics data at a single-cell level, including transcriptomics, epigenomics, and proteomics, holds great promise for unraveling the complex regulatory networks driving cancer cell behavior [17]. By combining these complementary datasets, researchers can gain a comprehensive understanding of the molecular mechanisms underlying tumor heterogeneity, paving the way for more effective and personalized cancer therapies.

## 4. Applications of Single-Cell Sequencing for Genomic Profiling in Human Cancer Cells

Single-cell sequencing (SCS), especially single-cell DNA sequencing (scDNA-seq) technologies, have impacted cancer analysis through the genomic profiling of single cells in the tumor and cancer. However, unlike traditional genomics, single-cell genomics targets intratumoral heterogeneity and microenvironmental elements involved in the response and resistance to treatment, providing a broader picture of cancer-related processes [36]. Here, we describe, in detail, some of the applications of scDNA-seq related to cancer:

(a) Tumor Heterogeneity and Clonal Evolution

Single-cell DNA sequencing has proven to be useful for the interrogation of intratumoral heterogeneity and clonal evolution in a plethora of malignancies. Studies pointed out that tumor heterogeneity is due to clonal evolution, creating a fractal pattern of the primary and secondary main clones, subclones, and single cells, raising issues on the best sampling strategy for tumor sequencing for clinical use [37] (Rajan et al., 2023). A great example is the use of single-cell DNA amplicon sequencing to track clonal heterogeneities of B-Cell Acute Lymphoblastic Leukemia (B-ALL) at diagnosis and during the course of chemotherapy treatment to investigate clonal evolution profiles in response to therapy [38].

The ability of the scDNA-seq method has recently been demonstrated to track clonal evolution and identify dynamics of oncogenic cells in cancer progression and treatment [39]. It is important to emphasize the applicability of single-cell DNA sequencing, where it is possible to directly obtain clonal genotypes at the cellular level and detect branches in clonal evolution, demonstrating that this approach is superior to other massive sequencing methods, such as bulk sequencing, for studying clonal evolution [40]. Rajan et al. (2023) analyzed, on 10 tumor samples and 12,019 tumor cells, intratumoral genomic heterogeneity and the evolution of somatic copy number alterations (SCNAs) in structurally complex osteosarcoma genomes. In this study, they described a genomic homogeneity on these, with surprising remarkably conserved profiles of SCNA with a limited variability among the subclones; also, they found whole-genome duplication (WGD) in many tumors, and these appear to be a mechanism to mitigate the effects of genetic deletions. The most genomic alterations in tumors were acquired early in the oncogenic process and remained stable over time, both in tumor progression and in response to therapy; this suggests an early catastrophic event, rather than sustained genomic instability as the primary cause of the tumor structural complexity. And these tumors exhibited remarkable stability in their SCNA profiles from diagnosis to relapse, indicating that there is no significant evolution in response to treatments [37].

(b) Identification of Rare Mutations

scDNA-seq has become a powerful tool in the identification of rare mutations in cancer, offering a high-resolution approach to uncover genomic complexity and compound mutations within individual cells [41]. Examining cells individually provides the ability to reveal diverse evolutionary trajectories between primary and metastatic tumor cells, yielding insights into genetic heterogeneity within cancer populations [42]. scDNA-seq has been instrumental in revealing the depth of genomic complexity and compound mutations within tumors, providing insights into the unique mutational profiles of individual cells [41]. This technique has proven to be valuable in the study of somatic mutations, allowing the detection of rare mutations that may go undetected with bulk sequencing methods, [43]. Gráf et al. (2021) illustrate how single-cell analysis can be used in conjunction with next-generation sequencing to identify the mosaicism of BRCA2 mutations and decode the cellular processes underlying murine tumorigenesis [44]. Furthermore, this strategy improves the sensitivity of the rare mutation detection of limited tumor cells by integrating laser capture microdissection (LCM) and NGS technologies, which shed light on BRCA1/2 mutations and the process of tumorigenesis.

(c) Drug Resistance and Mechanisms

scDNA-seq is a powerful tool that helps understand the resistance that occurs against cancer drugs. The analysis of individual cells offers information about the heterogeneity within tumors and reveals which subpopulations can generate resistance to treatment [45]. It has been shown that cancer stem cells, a small fraction of the tumor cell population, can cause progression, metastasis, and drug resistance [46]. Therefore, it is extremely important to identify and target these specific cell populations to improve treatment results.

Another exemplary case is the study by Lee et al. (2022). In this study, they employed a scDNA-seq approach to study clonal heterogeneity and clonal evolution in two patients with myelodysplastic syndrome (MDS) refractory to hypomethylating agents (HMAs). Importantly, HMAs are the mainstay of treatment for MDS; however, in most patients, resistance to treatment and transformation of the disease into acute myeloid leukemia (AML) was observed. Two patients with HMA resistance and progression to AML were studied. By bulk sequencing, different single-nucleotide variations (SNV) or insertions and deletions (INDELs) are detected in these patients, but using the scDNA-seq approach, rare cell clones with mutations that cannot be detected by bulk sequencing were detected, identifying pathogenic copy number variation (CNV) of GATA2, DNMT3A, and TET2, which are associated with resistance against HMA, but these CNVs could be coupled with small SNVs or INDELs of FLT3 and IDH2; all of these mutations work together, having an impact on disease progression and drug resistance [47].

Likewise, scDNA-seq also helps to discover and elucidate the mechanisms involved in these resistance events, as in the case of quizartinib where on- and off-target mechanisms of resistance that can preexist therapy were identified. In this study, they analyzed over 103,000 cells from 16 timepoints across eight patients, identifying pathogen variants undetected by bulks. In this work, they identified the FLT3 tyrosine kinase domain (TKD) as a primary mechanism of resistance to quizartinib. The on-target resistance mechanism is the kinase domain (KD) mutation; seven of eight patients developed at least one additional mutation in the FLT3 kinase domain, most commonly at the D3895 locus. These KD mutations can occur on the native FLT3-ITD (internal tandem duplication)-negative gene, in a cis orientation with FLT3-ITD [48].

(d) Detection and Diagnosis of the Presence of Cancer

scDNA-seq has become a powerful tool to aid and complement cancer diagnosis by providing information on the genetic heterogeneity and molecular characteristics of tumors at the single-cell level. Circulating tumor DNA (ctDNA) has been recognized as a valuable biomarker for the molecular diagnosis and monitoring of cancer progression through blood samples [49]. Single-cell sequencing techniques, especially scWGS, have enabled the detection of circulating tumor cells (CTCs) for liquid biopsy-based diagnosis of multiple cancers, allowing the identification of genuine CTCs through concordant copy number alteration profiles [50].

Wang et al. (2022) presented a molecular algorithm based on single-cell genomics for early cancer detection [51]. The study uses single-cell sequencing to examine somatic copy number alterations (CNAs) or oncogenic driver mutation profiles at the single-cell level to confirm cellular malignancy. The application of these technologies has also been seen in pancreatic cancer, significantly improving the sensitivity and specificity of cancer cell detection, allowing the analysis of rare cancer cells, circulating tumor cells, and metastatic cells [52,53].

Beyond its transformative impact on cancer research, SCS has demonstrated remarkable potential in other fields of biology and medicine. For instance, in neurology, SCS is increasingly used to study somatic mutations in brain cells, shedding light on the genetic underpinnings of neurodegenerative diseases such as Alzheimer’s [47]. In the field of autoimmune diseases, SCS aids in identifying genetic variations within specific immune cell populations, providing valuable insights into disease mechanisms and potential therapeutic targets [54]. Similarly, in reproductive medicine, SCS plays a critical role in assessing genetic mosaicism in embryos, which is pivotal for fertility studies and prenatal diagnostics [55]. Additionally, SCS, especially scRNA-seq, has made significant strides in microbial genomics, enabling researchers to uncover genomic heterogeneity within microbial communities, which can inform strategies to combat antimicrobial resistance [56]. These diverse applications highlight the versatility of SCS and its potential to revolutionize fields beyond oncology.

## 5. Applications of Single-Cell Sequencing for Transcriptomic Profiling in Human Cancer Cells

SCS has emerged as a pivotal method for exploring gene regulatory networks (GRNs) and cellular dynamics [31]. Among these technologies, scRNA-seq provides a detailed view of transcript expression levels and patterns within individual cells across different subpopulations [57,58,59]. Here, we describe applications to sc-RNA related to cancer.

(a) Identifying cancer stem cells and rare cell populations

CSCs (cancer stem cells) are primitive, undifferentiated cells with characteristics similar to normal stem cells [60,61]. However, CSCs are biologically characterized by self-renewal, multi-directional differentiation, and infinite proliferation, inducing antitumor drug resistance and metastasis [62]. Pan et al. characterized and identified primary and metastatic duct renal cell carcinoma (CDRCC), observed CSC-specific markers to be correlated with the poor prognosis of CDRCC, and pinpointed inhibitors for effectively targeting CSCs as potential therapeutic strategies for CDRCC [62].

Other CSC malignancies such as pancreatic cancer have been studied by Ren et al. (2021), specifically pancreatic duct adenocarcinoma (PDAC), which is an aggressive and lethal malignancy [60]. This study revealed the heterogeneity of ductal cells and identified nine main clusters, endocrine, acinar, endothelial, ductal, myeloid, fibroblast, pericyte, T, and B cells, which were then reclustered into six different clusters. Most enriched clusters in tumor tissue exhibited remarkably high copy number variation (CNV) levels. The expression of specific marker genes was found to be specific to each cluster type. For example, in cluster 1, acinar epithelial-related genes were expressed in PRSS1 and CLPS [63]; heat shock protein-related genes expressed in cluster 6 were involved in protein transport and folding of ductal cells [64]; and clusters 3, 4, and 5 primarily expressed genes related to the proliferation and potential invasion of cells. Single-cell trajectory analysis showed that pancreatic duct cells originated from clusters 1 and 6, transitioned to cluster 2, and finally evolved into clusters 3, 4, and 5. This study also identified 202 cancer-related genes (CRGs), including 140 upregulated CRGs and 62 downregulated CRGs, and established a relationship between key genes and patient survival. The frequency of mutation events in the high-risk cohort was significantly higher. Kras mutation indicated poor survival in patients. LY6D and MET were significantly more highly expressed in tumor tissues than in normal pancreatic tissues.

(b) Determining heterogeneity within a cell population

Heterogeneity between different malignant cells is one of the fundamental characteristics of almost all human cancers [65,66,67]. By measuring transcriptomes at the single-cell level, scRNA-seq enables the identification of cellular heterogeneity in far greater detail than conventional methods [68]. Furthermore, scRNA-seq can be used to identify specific modes of gene expression authorized for the elucidation of molecular mechanisms underlying tumor migration and invasion [67,69]. Also, constructing gene regulatory networks (GRNs) from scRNA-seq data is used to explore intratumoral heterogeneity and can elucidate the critical genes involved in cancer development [67]. For instance, Wouters et al. (2020) used single-cell transcriptomics in combination with GRNs and trajectory inference to study 10 melanoma cultures that have been used to map the gene regulatory landscape of relapsed melanoma [67,70].

(c) Tumor immunology

The immune system is composed of a complex hierarchy of cell types that protect the organism against disease and maintain homeostasis [68]. Understanding how the immune system affects cancer development and progression has been one of the most challenging questions in immunology [71]. Identifying the heterogeneity of immune cells is the key to understanding the immune system. Advanced scRNA-seq technologies are revolutionizing our ability to study immunology. Recent studies from Hui et al. used scRNA-seq to reveal how a neoadjuvant PD-1 blockade combined with chemotherapy remodels the tumor microenvironment in non-small-cell lung cancer. They identified significant remodeling of immune cells within the tumor, including the expansion, activation, and phenotypic alterations of cytotoxic T cells, CD16+ natural killer (NK) cells, and regulatory T cells (Tregs) following therapy [72].

(d) Cancer progression, drug development, and cancer treatment

There is an increased interest in the potential clinical application of single-cell techniques reflected in collaborative initiatives like LifeTime. LifeTime aims to understand the complex behavior of human cells during disease progression and analyze their response to the therapy, all at single-cell resolution [73,74]. To achieve this, further development and integration of multi-omics methods is urgently needed [74].

ScRNA-seq has enabled the identification of molecular pathways that allow the prediction of survival [75,76], response to therapy [76,77], likelihood of resistance [76,78,79] and candidacy for alternative intervention [76,80].

Recently, an increasing number of clinical trials have been integrating RNA-seq in their design with various objectives: either for the biological description of the effect of treatments or with the intent to treat patients [57]. Single-cell genome-plus-transcriptome sequencing is also a valuable tool for studying the efficacy and safety of genome editing in germline therapy. Genome editing of human embryos or germ cells provides the means for introducing heritable genetic alterations, which may reduce the burden of genetic disease in specific familial situations [81,82]. For instance, Vishnubalaji and Alajez (2023) used computational algorithms to decipher the cellular composition of various breast cancer (BC) subtypes, including estrogen receptor-positive (ER+), HER2+, ER+HER2+, and triple-negative (TNBC) subtypes [83]. They analyzed transcriptomic data from 49,899 single cells, derived from 26 BC patients, and integrated differentially expressed genes with CRISPR-Cas9 perturbational gene effect data from the Achilles project [84]. The key discoveries include ER+ BC: 13 targets, with RPS4X, RPL34, and VMP1 being more effective than ESR1; HER2+ BC: 44 targets, with some stronger than ERBB2, with enriched processes in mRNA decay and protein targeting; and TNBC: 29 targets, which are enriched in processes like protein targeting and mRNA processing. Overall, these findings point to new potential targets for treating each subtype.

Other studies use tumor model systems exposed to treatment or direct longitudinal sampling of patient tumor specimens before and during treatment to analyze single-cell genome-plus-transcriptome sequencing to understand the genetic subclones resistant to drug selection. Additionally, this will allow the study of how cells within these genetic subclones putatively apply cell plasticity to change their gene expression repertoire and adopt different phenotypic cancer cell states capable of surviving drug treatment and ultimately developing resistance [81,85]. These strategies would also help identify potential vulnerabilities in cancer cells, such as druggable targets and pathways involved in drug resistance [81].

**Figure 3:**
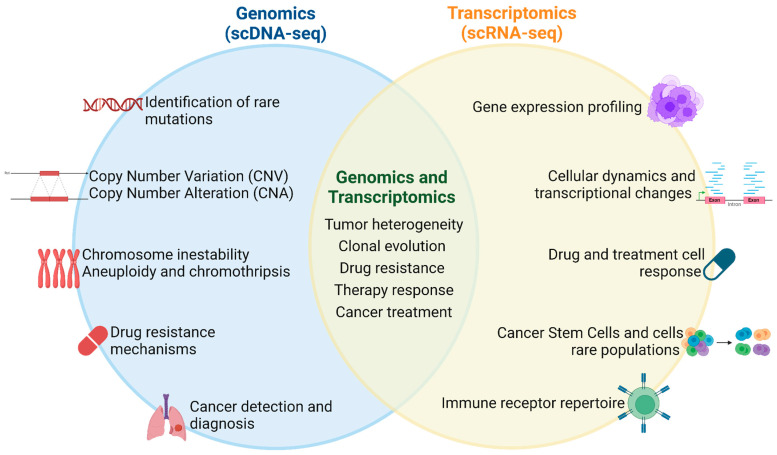
Applications of single-cell sequencing for genomic and transcriptomic profiling in human cancer cells. Applications that are carried out by both technologies, independently or integrated, are found in the middle. Created in www.BioRender.com. Accessed on 30 October 2024.

Single-cell DNA sequencing has significantly enhanced our understanding of cancer in human cells by enabling the tracking and reconstruction of clonal evolution, the detection of rare mutations, and the study of drug resistance mechanisms. Furthermore, its integration with single-cell RNA sequencing enhances diagnostic precision and provides a comprehensive molecular characterization of tumor cell populations, tumor heterogeneity, and treatment responses (Figure 3).

## 6. ‘Co-Presence’ and ‘Phenotypic Association’ Capability of scSeq Technology

The ‘co-presence’ functionality of SCS technology refers to its ability to identify the various genomic features or variations concurrently present within each cell analyzed [17]. For example, it can detect the simultaneous presence of multiple or single mutations [86,87], copy number alterations [88,89], and structural variants [90] within the same single cell. This capability provides insights into the interplay between different genomic aberrations and their potential combined effects on cellular processes or disease development [91]. By capturing the co-occurring genomic variations within individual cells, researchers can better understand the heterogeneity and complexity of genomic landscapes, particularly in diseases like cancer, where cells can harbor diverse combinations of mutations and other alterations. Furthermore, the ability to detect co-presence in SCS enables scientists to concurrently identify the manifestation of both genetic alterations and epigenetic changes within each cell analyzed [92] as well as uncover widespread variation among malignant cells concerning their cellular identity and developmental staging [93]. Therefore, co-presence is a crucial capability of SCS that enables many of its unique applications, revolutionizing our understanding of genomics and disease development.

Conventional sequencing methods, which analyze entire cell populations, often face significant limitations. One such limitation is their inability to detect the simultaneous presence of multiple mutations within individual cells. This drawback results in a loss of information about the cellular diversity within the population [94]. In contrast, single-cell omics offer a superior approach to unraveling cellular diversity. SCS, in particular, preserves this information, allowing for a more holistic study of cells. Interestingly, studies have begun to use both single-cell sequencing and multi-omics (bulked sequencing) datasets to analyze the molecular diversity of hepatocellular carcinoma (HCC) across inter- and intratumor levels [95]. This approach exemplifies how we can enhance the understanding of co-presence in cell studies by integrating cutting-edge and traditional technologies.

The complexities surrounding co-presence and phenotypic association analysis encompass the widely recognized technical noise, the intricacies of data analysis, and the necessity for integrative approaches [96]. Regardless of these challenges, SCS persists as a potent methodology for comprehensively investigating co-presence and phenotypic association in cellular entities.

## 7. Immune Cell Response in Tumor Microenvironment Using SCS

Beyond the important roles immune cells play in determining the immune responsiveness of tumors, they are also important direct targets of immunotherapeutic strategies. It is thus pivotal to identify molecular mechanisms that modulate immune responsiveness and therapeutic performance of the tumor microenvironment (TME) immune cells. These mechanisms involve non-genetic events that pose significant regulatory elements to be characterized. For instance, Huang et al. highlighted the role of microvascular invasion-related malignant cells, which are driven by MYC pathway activation and MIF signaling within the TME of hepatocellular carcinoma (HCC). Their findings underscore how specific malignant subpopulations interact with immune cells, shaping prognosis and immune responsiveness [97]. At the same time, cellular transition trajectory analysis in single-cell epigenomes further demonstrates that epigenetic features do not always directly predict future cell fate or gene activities but are reciprocal to transcriptional dynamics. Identification and decoding of the epigenetic features associated with unique TME states and functions in vivo would thus require extensive studies of the single-cell TME epigenomes and their interplay with the transcriptional landscape [98]. Because of these important roles TME immune cells play in predicting the prognosis and therapeutic performance of distinct human cancer types, their molecular properties urgently need to be characterized with personalized precision medicine, so that therapeutic strategies to efficiently modulate within TME immune cells can be designed [99].

It has been documented that various immune cells that are part of the TME can play important roles in promoting or inhibiting tumor development and metastasis. These cells, collectively called tumor-infiltrating immune cells 1 (TIICs), are also significantly associated with clinically relevant features, such as patient survival and response to immunotherapy. Efforts aiming to understand the molecular characteristics of immune cells, especially those within the TME, have thus gained significant traction. To date, single-cell genomics has emerged as a widely used tool to study TIICs, has identified various subpopulations of T cells and myeloid cells, and has shown spatial, differentiation, and functional heterogeneity within the TME with the use of Multimodal Intersection Analysis (MIA) and Spatial Transcriptome Deconvolution (STD) [100]. For instance, Zhang et al. (2021) demonstrated age-related differences in the immune landscape of melanoma-bearing mice, where older mice exhibited a higher proportion of cytotoxic CD8+ T cells and fewer exhausted cells, contributing to enhanced antitumor immunity. This highlights the importance of dissecting immune cell subpopulations at the single-cell level to better understand their role in tumor progression and response to immunotherapies [101].

Similarly, Chen et al. (2024) demonstrated that the upregulation of HMGB2 shapes the immunosuppressive microenvironment in HCC, correlating with exhausted T cells and poorer clinical outcomes. This underscores how epigenetic and transcriptional regulation of factors like HMGB2 can hinder immune cell functionality and drive resistance to immunotherapy, making it a key target for therapeutic intervention [102]. Multi-omics technologies that provide single-cell data at the transcriptional/genomic, epigenetic, proteomic, and other levels have been pivotal in exploring the states and functions of TME immune cells. These have shown that epigenetic dynamics have a prominent influence on the cellular status of TME immune cells. Furthermore, parallel profiling of chromatin state and gene expression in single cells revealed that chromatin modifications could be predictive of cell state dynamics and that cellular transcriptomes might not always reflect their epigenetic status. Myeloid-lineage immune cells are also influenced by epigenetic chromatin modifications, which can directly control their TME functions.

(a) Tumor microenvironment

Tumors are highly complex entities composed of heterogeneous tumor cells, stroma cells, and immune cells [103]. Intertwined biological processes between different types of cells facilitate tumorigenesis, tumor progression, and resistance to therapy, as well as contribute to tissue homeostasis maintenance of normal physiological processes. Consequently, for effective therapeutic intervention, it is crucial to understand the underlying cellular and molecular knowledge and their roles in orchestrating the tumor microenvironment [104]. The constituents of the TME and their role in tumorigenesis and in response to therapy encompass a rapidly evolving field, thanks to cutting-edge methodological advances. Single-cell analysis has facilitated the understanding of immune cell responses in the TME and has allowed researchers to study the functional and molecular aspects of cancer immunologists in greater detail. For instance, Pires et al. (2020) demonstrated how T cells drive ECM remodeling and CSC reduction in fibrosarcomas, revealing how immune activity in the TME directly impacts tumor rejection and progression [105]. A thorough understanding of immune cell activity in the TME is essential in developing newer drugs and treatment options for cancers.

(b) Cellular Components of Tumor Microenvironment

These immune cells interact with each other and with cancer cells, secreting a variety of soluble factors and contributing to tumor initiation, progression, and invasion. The function of these cellular components and the tissue-specific microenvironment are deregulated in TME, with pro-tumorigenic and antitumorigenic activities being intertwined.

The TME consists of non-cancerous cells, their associated cell products, and an extracellular matrix within and around the tumor mass. The cellular components of the TME are macrophages, granulocytes, mast cells, myeloid-derived suppressor cells, dendritic cells, T cells, B cells, natural killer cells, cancer-associated fibroblasts, mesenchymal stromal cells, endothelial cells, pericytes, and nerve cells. These cells perform various functions such as immune response, cell signaling, and extracellular matrix remodeling, which are crucial for tumor growth and progression. For instance, Anderson and Simon highlighted that macrophages, especially M2 macrophages, along with myeloid-derived suppressor cells (MDSCs), contribute to immunosuppression by secreting cytokines such as IL-10 and TGF-β. These cytokines activate key signaling pathways like NF-κB and STAT3, facilitating tumor growth and immune evasion. Additionally, cancer-associated fibroblasts play a significant role in ECM remodeling, supporting metastasis and tumor invasion [106]. Alcantara et al. (2023) further demonstrated that targeting STAT3 activation in MDSCs through CpG-STAT3 antisense oligonucleotide (ASO) therapy significantly reduces immunosuppression and enhances T-cell activity in renal and bladder cancer. This combination therapy, when used with anti-PD-1, improved tumor control by reducing MDSC-driven immune resistance and promoting tumor regression [107]. Li et al. (2020) emphasize that tumor-associated macrophages (TAMs) and MDSCs, along with cytokines like IL-6 and TGF-β, contribute to chronic inflammation and immunosuppression within the TME. Targeting these cells and pathways could improve cancer therapies by disrupting the immunosuppressive TME [108]. Using single-cell sequencing and complementary technologies such as spatial transcriptomics helps to combine sequencing data with spatial information to address the research of spatial distribution in the TME.

(c) Overview of tumor microenvironment at single-cell resolution

The accessibility of single-cell sequencing to mixed-cell populations in a non-perturbing manner has made it an attractive technique to rationalize the dynamics of cell heterogeneity in the TME. Due to their relatively low assay cost and high-throughput performance compared with imaging technologies, single-cell sequencing has been widely applied to study various types of tumors and mouse models. While traditional SCS lacks inherent spatial resolution, its integration with spatial transcriptomics methods, such as Slide-seq, MERFISH, and 10X Genomics Visium, enables the mapping of single-cell transcriptomic profiles onto tissue architecture. This approach allows researchers to correlate gene expression patterns with specific tumor regions, revealing the spatial organization of tumor cells and immune interactions within the TME. Additionally, computational tools like Seurat and STutility can infer spatial localization by integrating single-cell RNA-seq data with spatial reference atlases, further enhancing our understanding of cellular heterogeneity in the tumor microenvironment. Moreover, SCS reveals signaling interactions among different cell types, potentially uncovering novel factors driving the initiation and progression of cancers besides uncovering novel cell subsets with unknown functions [109,110].

From a translational perspective, single-cell sequencing provides vast advantages over the traditional bulk RNA-seq assay since the former could identify the complex heterogeneity in not only the cancer cells themselves but also, historically, in stroma cells, providing opportunities as well as challenges for developing novel cancer treatments. Single-cell sequencing provides comprehensive views on the statics and dynamics of the cell composition in the TME along with their spatial distributions, which not only facilitate the discovery of potential biomarkers in a clinical routine but also advances researchers from bench to bedside, which would subsequently benefit cancer patients in the future [111,112,113].

A central question addressed by single-cell sequencing of the tumor microenvironment is the cellular heterogeneity among stromal and immune cell types. Importantly, single-cell sequencing allows for defining cell populations at higher resolution than is possible by bulk sequencing, offering the potential identification of biomarkers that would be otherwise diluted when bulk material is used. To this end, single-cell sequencing of dendritic cell subsets is a perfect test case. Dendritic cells are generally a rare cell type in the whole blood (0.36%), and single-cell technology could offer a better representation of the subsets such as plasmacytoid dendritic cells (pDCs). The ability to define these dendritic cell subsets would have great implications in allowing personalized treatment of cancer through the application of active immunotherapies. Recently, single-cell RNA sequencing has been used to define dendritic cells, monocytes, and other diverse cell populations in triple-negative breast cancer (TNBC) and murine melanoma tumors. For instance, Gao et al. (2021) utilized scRNA-seq to reveal the heterogeneity of monocyte-derived dendritic cells (moDCs) and type-2 conventional dendritic cells (cDC2s). Their study identified seven distinct DC subtypes, with functional differences in antigen processing and immune regulation, especially during maturation. This demonstrates the utility of scRNA-seq in uncovering novel immune cell subsets and their potential roles in shaping tumor immunity, offering insights into how these cell populations could be targeted for immunotherapy [114]. scRNA-seq allows us to gain further insight into the spatial localization of tumor and stromal cells in the tumor microenvironment compared to bulk RNA sequencing technology. Distinct cellular localization within different parts of the tumor microenvironment, for example, within the tumor, invasive margin, and the center of the tumor, or between epithelial and stromal compartments, may allow for a dissection of the distinct functions and mechanisms of tumor-associated cells [115,116,117].

## 8. Role of Single-Cell Data Analysis Technologies in Cancer Therapy

Cancer is a complex and heterogeneous disease characterized by diverse cellular populations within a tumor and dynamic interactions between cancer cells and their microenvironment. This heterogeneity poses significant challenges in understanding disease mechanisms and developing effective treatments. The advent of single-cell data analysis technologies has revolutionized our ability to dissect the complexities of cancer at an unprecedented resolution, unveiling new insights into tumor biology and paving the way for personalized cancer therapy. The increase in SCS data and methodologies for wet-lab applications has been paralleled by advancements in data analysis tools to interpret these data [13,118].

The data analysis options available for a particular biological sample are largely dictated by the sequencing technique used to generate the raw data and depend on the research question and study objectives. The type of cell isolation employed in a study is closely aligned with its performance regarding the capture efficiency and purity of the target cells, which directly affect the output data and consequently the data analysis approach [119,120]. For example, a droplet-based method is particularly effective for characterizing tissue composition, as it enables the capture of a substantial number of cells [121].

**Figure 4:**
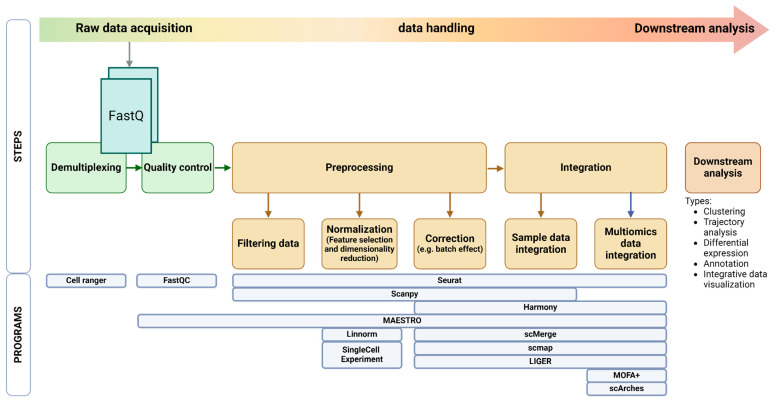
Overview of common tools used in scRNA-seq data handling workflow. The workflow begins with raw data acquisition in FastQ format, followed by demultiplexing and quality control to ensure reliability. It then transitions to the preprocessing and integration stages. In preprocessing, data undergo filtering to eliminate low-quality cells, normalization to address technical variations, and correction for batch effects. The integration phase combines data from different samples and/or incorporates multi-omics data for a comprehensive analysis of cell populations, depending on the study. Finally, the workflow culminates in downstream analysis, encompassing clustering to identify distinct cell populations, trajectory analysis to infer developmental pathways, differential expression analysis for gene variation, annotation for cell-type identification, and interactive data visualization to interpret complex datasets. This systematic approach provides a simplified overview of the steps used for processing and analyzing scRNA-seq data. Finally, the bottom of the image highlights the most commonly used programs for each step of the suggested workflow. Created in www.BioRender.com. Accessed on 5 February 2025.

Single-cell data analysis generally involves distinct preprocessing and integration stages. Preprocessing focuses on preparing individual datasets through demultiplexing, quality control, and data filtering and normalization. Integration then combines these datasets, using methods like data alignment and correction to account for technical variations (e.g., batch effects) and/or integrate data from different omics (Figure 4). Feature selection and dimensionality reduction are often performed after integration to facilitate downstream analyses like clustering and trajectory inference. The pipeline choice can have a comparable or even greater impact on the ability to detect biological signals in scRNA-seq data compared to increasing the cell count from 96 to 384 cells analyzed [122].

(a) Preprocessing and Integration

The analysis of single-cell data begins with robust and effective data handling and integration. CellRanger is a key pipeline that primarily carries out the initial step of preprocessing raw single-cell RNA-seq by performing demultiplexing, alignment, and gene expression quantification [14]. It uses these barcodes and UMIs to find low-quality cells and identify reads originating from the same cell [123]. It is also known that it can cluster similar or the same cells into non-overlapping groups. Some tools merge data handling and integration with the subsequent analysis for a multimodal approach [124]. Seurat is a great example of a multimodal analysis tool that enables researchers to conduct further analysis using data handling and integration [125]. However, there are tools dedicated only to data handling experiments, like SingleCellExperiment [126,127], which include specialized methods to store and retrieve spike-in information, dimensionality reduction coordinates, and size factors for each cell, along with the usual metadata for genes and libraries. This flexible data representation is compatible with the Bioconductor ecosystem, allowing seamless integration with other analysis tools and pipelines. Additionally, tidySingleCellExperiment bridges the gap between SingleCellExperiment objects and the Tidyverse ecosystem [128], promoting reproducible and efficient data handling and integration workflows.

Due to the difference in experimental methods and computational analysis in SCS, directly comparing cell identities between different experiments can be challenging. For this issue, tools like scmap [129] address this by allowing users to integrate cell types or individual cells in different experiments. Similarly, LIGER [130] can be used to compare and contrast across experimental batches, individuals, sex, tissues, and species and is particularly useful for handling different modalities like RNA-seq and ATAC-seq.

Unlike the single-cell experiment, scmap offers further data exploration (downstream analysis) to identify clusters, find shared gene markers, compare clusters, and visualize clusters and gene expression. Similarly, scMerge [131] offers a robust method for merging multiple batches of single-cell RNA-seq data, accounting for potential batch effects and enabling integrated analyses across diverse datasets. MAESTRO [132], besides carrying out quality control, normalization, and filtering of single-cell data, specializes in the integration of a single-cell transcriptome and regulome for evaluating scATAC-seq clustering, automatic cell-type annotation, and integration between scRNA-seq and scATAC-seq.

Moreover, many specialized tools analyze specific modalities. DropletUtils [133] facilitates the analysis of droplet-based single-cell data, while zellkonverter [134] enables interoperability by converting between Python and R environments, allowing seamless data transfer between these platforms. Furthermore, data normalization and transformation methods, such as Linnorm [135], play a crucial role in ensuring data quality and comparability across samples. Linnorm provides robust normalization and transformation of data based on linear models and normality assumptions, enabling accurate downstream analyses. It preserves biological variations in scRNA-seq data and removes technical noises simultaneously.

Many studies utilize a multi-omics approach for cancer research, highlighting the importance of accurately integrating diverse data types for effective analysis. By combining transcriptomics, epigenomics, and proteomics, researchers can gain a comprehensive understanding of cellular states and the regulatory mechanisms that drive them [136,137,138]. Tools like MOFA+ [139] and scArches [140] enable the integration of diverse omics data, revealing shared and unique features across different molecular layers. Spatial analysis techniques, such as spatialHeatmap [141], spicyR [142], and SpatialExperiment [143], facilitate the exploration of spatial patterns and heterogeneity within tumor samples, providing insights into the tumor microenvironment and its influence on cancer progression and treatment response.

(b) Clustering and downstream analysis

Identifying distinct cell populations and subgroups within heterogeneous tumor samples is a critical step in understanding cancer biology. Clustering algorithms like bluster [144], celda [145], and SC3 [146] group cells based on their transcriptome or genomic profiles, enabling the identification of distinct cell populations. Differential analysis methods, such as DEsingle [147] and distinct [148], identify genes, pathways, or molecular features that are differentially expressed or regulated between cell populations or experimental conditions, providing insights into the molecular drivers of cellular heterogeneity.

Several key algorithms are commonly employed in single-cell clustering analysis, with the Leiden algorithm standing out as a refined and improved version of the Louvain algorithm. It has demonstrated superior performance compared to other clustering methods for single-cell RNA-seq data analysis [149]. As the Louvain algorithm is no longer actively maintained, the Leiden algorithm has become the preferred choice. Widely used single-cell analysis tools, such as Seurat (R-based) and Scanpy (Python-based), have integrated both algorithms for clustering applications. The Leiden algorithm follows a structured process consisting of three key phases: (1) local movement of nodes to optimize modularity, (2) refinement of the partition to improve cluster accuracy, and (3) aggregation of the network based on the refined partition, with the non-refined partition serving as the initial input for the aggregated network [149].

As mentioned before, there are well-known multimodal tools like Seurat [125] (Hao et al., 2024) and Scanpy [124] (Wolf et al., 2018) that enable quality control, analysis, clustering, and differential expression capabilities for scRNA-seq data, facilitating the identification and interpretation of cellular heterogeneity. Tools like SCENIC [150] (Aibar et al., 2017) infer active transcription factors and gene regulatory networks, providing insights into gene regulation within tumor cells. CopyKAT [151] (Gao et al., 2021) and InferCNV (inferCNV of the Trinity CTAT Project) specialize in inferring genome-wide copy number variation profiles from scRNA-seq data, a critical aspect of cancer genomics. Other recently used tools, such as non-linear dimensional reduction (UMAP), cluster cells with a graph-based clustering approach [152,153], and SingleR performs cluster definition plus annotation [154].

(c) Single-Cell DNA and Whole-Genome Sequencing (scWGS)

Single-cell DNA-sequencing (scDNA-seq) and whole-genome sequencing (scWGS) techniques offer unprecedented insights into genomic alterations, such as copy number alterations, copy number variations, and clonal heterogeneity within tumors. Here, we present some tools for the downstream data analysis of scDNA-seq. The tool Ginkgo [155] identifies copy number variations, and CHISEL [156] infers copy number alterations including whole-genome duplications (WGDs), while PyClone [157] and SCCNV [158] elucidate clonal populations and their evolutionary trajectories. SCcaller [159] and SCCNV identify single-nucleotide variations and copy number variations, respectively, and SCITE [160] is a tool designed to infer the evolutionary history of tumors using noisy and incomplete mutation profiles of single cells from scDNA-seq data.

The integration of single-cell data analysis technologies has revolutionized our understanding of cancer biology, enabling the exploration of tumor heterogeneity, clonal evolution, and cellular interactions within the tumor microenvironment. As single-cell technologies continue to advance, data analysis tools will play a crucial role in leveraging the wealth of information contained within these datasets, ultimately paving the way for personalized and targeted cancer therapies tailored to individual patients’ tumor profiles.

## 9. Emerging Technologies and Future Directions in scWGS in Cancer Biology

The rise in single-cell whole-genome sequencing (scWGS) has transformed cancer biology, offering detailed insights into the molecular landscapes of cancer cells. Deep learning techniques have proven highly effective in analyzing the vast and complex data generated by scWGS [161,162]. These methods have outperformed traditional computational approaches in various domains [163,164]. In cancer biology, deep learning has facilitated the study of tumor cell heterogeneity, uncovering gene expression patterns and regulatory networks governing cell behavior [162,165].

Artificial intelligence (AI) has emerged as a powerful tool in scWGS analysis, with machine learning (ML) algorithms excelling at extracting valuable insights from high-dimensional and complex data [166]. In cancer research, AI has contributed to the development of prognostic models, biomarker identification, and characterization of the tumor microenvironment [167,168]. For example, Chen et al. (2023) used AI to construct a communication signature derived from cancer-associated fibroblasts (CAFs), enabling the stratification of clear-cell renal-cell carcinoma patients based on their immune profiles and potential response to immunotherapy [169]. Similarly, Kang et al. (2024) employed AI techniques to identify tumor-associated macrophage subpopulations in prostate cancer, revealing subpopulations that may facilitate tumor progression by enhancing immune evasion and altering the tumor microenvironment, which can lead to therapeutic challenges in tumor progression and drug resistance [170].

Deep learning, a branch of AI, has emerged as a transformative force in scWGS, offering algorithms that can integrate multi-omics data, including genomics, transcriptomics, and proteomics, to unravel the complexities of tumor heterogeneity and identify cancer subtypes [171,172]. Danishuddin et al. (2024) explored the applications of deep learning in cancer genomic and proteomic studies, highlighting its potential to improve patient diagnosis, prognosis, and treatment strategies [173]. In glioma research, Luo et al. (2023) demonstrated the utility of deep learning in tumor segmentation, diagnosis, grading, and characterization of the tumor microenvironment, paving the way for personalized treatment approaches [174]. As scWGS continues to evolve, deep learning will play a role in extracting meaningful insights from multi-omics data, driving the development of precision oncology.

In the future, developing technologies for single-cell analysis in cancer is crucial. Techniques in addition to DNA-based and RNA-based methods, such as scEpigenetics, sc proteomics, sc metabolomics, sc CRISPR technologies, and sc multi-omics technologies, may be particularly helpful in advancing this field. Additionally, the advancements in machine learning, AI, and data analysis will further enhance our understanding and capabilities in cancer biology and its implications. Consequently, more studies focusing on areas such as prostate cancer, breast cancer, cervical cancer, and solid tumors, especially for complex matrices that are difficult to understand, would be beneficial in the future in terms of diagnosis, microenvironment, understanding of the existing pre- and post-resistance, and better targeted and efficient therapies, which should all be supported by a robust bioinformatics analysis that will enhance these benefits through the incorporation of cutting-edge technologies such as artificial intelligence.

The integration of these tools into cancer profiling has greatly enhanced our ability to understand tumor complexity, genomic instability, and clonal evolution. This comprehensive understanding of tumor complexity allows for more accurate predictions for treatment responses, the identification of resistance mechanisms, and the development of more personalized and effective therapeutic strategies, paving a way for precision oncology.

## 10. Conclusions

Single-cell sequencing (SCS) has redefined our understanding of cancer biology, providing unprecedented insights into tumor heterogeneity, clonal evolution, and the complex interactions within the tumor microenvironment. This technological breakthrough has enabled researchers to analyze the genomic, transcriptomic, and epigenomic landscapes of single cells and uncover the complex cellular dynamics that drive cancer progression, metastasis, and treatment resistance.

The unique capabilities of SCS, particularly its accuracy in detecting rare genomic events, co-presence functionality for capturing multiple genomic features simultaneously, and its potential for phenotypic association potential, have opened new avenues for cancer research and precision medicine. These advances have facilitated the identification of rare cell populations such as cancer stem cells and elucidated the heterogeneous nature of tumors with unprecedented resolution.

Furthermore, the integration of SCS omics technologies, including scDNA-seq, scRNA-seq, scATAC-seq, and single-cell proteomics, has enabled a multidimensional view of cellular states and regulatory mechanisms in cancer. This integrative approach has enhanced our ability to decipher the complex interplay between genetic alterations, gene expression patterns, and epigenetic modifications that contribute to tumor initiation, progression, and therapeutic response.

In summary, the challenges regarding co-presence and phenotypic association analysis include the well-known technical noise, data analysis complexities, and the need for integrative approaches. Dropout events, amplification bias, and sequencing errors are common technical noises that compromise the data quality. Data analysis complexities stem from difficulties in accurate variant calling, interpreting cellular heterogeneity, and integrating genomic data with phenotypic traits, which will be discussed further in the subsequent section. Moreover, integrative approaches such as multi-omics integration, longitudinal studies, and functional validation add complexity but are essential to creating robust studies. Other issues like sample preparation, scalability, cost, statistical power in cohorts, and the need for standardized protocols also present significant hurdles. Despite these challenges, SCS remains a powerful methodology for delving deeper into the analysis of co-presence and phenotypic association in cells.

## References

[1] Navin N., Kendall J., Troge J., Andrews P., Rodgers L., McIndoo J., Cook K., Stepansky A., Levy D., Esposito D. (2011). Tumour Evolution Inferred by Single-Cell Sequencing. Nature.

[2] Suvà M.L., Tirosh I. (2019). Single-Cell RNA Sequencing in Cancer: Lessons Learned and Emerging Challenges. Mol. Cell.

[3] Patel A.P., Tirosh I., Trombetta J.J., Shalek A.K., Gillespie S.M., Wakimoto H., Cahill D.P., Nahed B.V., Curry W.T., Martuza R.L. (2014). Single-Cell RNA-Seq Highlights Intratumoral Heterogeneity in Primary Glioblastoma. Science.

[4] Tirosh I., Izar B., Prakadan S.M., Wadsworth M.H., Treacy D., Trombetta J.J., Rotem A., Rodman C., Lian C., Murphy G. (2016). Dissecting the Multicellular Ecosystem of Metastatic Melanoma by Single-Cell RNA-Seq. Science.

[5] Puram S.V., Tirosh I., Parikh A.S., Patel A.P., Yizhak K., Gillespie S., Rodman C., Luo C.L., Mroz E.A., Emerick K.S. (2017). Single-Cell Transcriptomic Analysis of Primary and Metastatic Tumor Ecosystems in Head and Neck Cancer. Cell.

[6] Han Y., Wang D., Peng L., Huang T., He X., Wang J., Ou C. (2022). Single-Cell Sequencing: A Promising Approach for Uncovering the Mechanisms of Tumor Metastasis. J. Hematol. Oncol..

[7] Hu Y., Shen F., Yang X., Han T., Long Z., Wen J., Huang J., Shen J., Guo Q. (2023). Single-Cell Sequencing Technology Applied to Epigenetics for the Study of Tumor Heterogeneity. Clin. Epigenetics.

[8] Loo J.F.-C., Ho H.P., Kong S.K., Wang T.-H., Ho Y.-P. (2019). Technological Advances in Multiscale Analysis of Single Cells in Biomedicine. Adv. Biosyst..

[9] Rognoni E., Watt F.M. (2018). Skin Cell Heterogeneity in Development, Wound Healing, and Cancer. Trends Cell Biol..

[10] Grün D., van Oudenaarden A. (2015). Design and Analysis of Single-Cell Sequencing Experiments. Cell.

[11] Hu P., Zhang W., Xin H., Deng G. (2016). Single Cell Isolation and Analysis. Front. Cell Dev. Biol..

[12] Gao C., Zhang M., Chen L. (2020). The Comparison of Two Single-Cell Sequencing Platforms: BD Rhapsody and 10x Genomics Chromium. Curr. Genomics.

[13] Svensson V., Vento-Tormo R., Teichmann S.A. (2018). Exponential Scaling of Single-Cell RNA-Seq in the Past Decade. Nat. Protoc..

[14] Zheng G.X.Y., Terry J.M., Belgrader P., Ryvkin P., Bent Z.W., Wilson R., Ziraldo S.B., Wheeler T.D., McDermott G.P., Zhu J. (2017). Massively Parallel Digital Transcriptional Profiling of Single Cells. Nat. Commun..

[15] Picelli S., Faridani O.R., Björklund A.K., Winberg G., Sagasser S., Sandberg R. (2014). Full-Length RNA-Seq from Single Cells Using Smart-seq2. Nat. Protoc..

[16] Wu S., Schmitz U. (2023). Single-Cell and Long-Read Sequencing to Enhance Modelling of Splicing and Cell-Fate Determination. Comput. Struct. Biotechnol. J..

[17] Evrony G.D., Hinch A.G., Luo C. (2021). Applications of Single-Cell DNA Sequencing. Annu. Rev. Genomics Hum. Genet..

[18] Macaulay I.C., Haerty W., Kumar P., Li Y.I., Hu T.X., Teng M.J., Goolam M., Saurat N., Coupland P., Shirley L.M. (2015). G&T-Seq: Parallel Sequencing of Single-Cell Genomes and Transcriptomes. Nat. Methods.

[19] Demaree B., Delley C.L., Vasudevan H.N., Peretz C.A.C., Ruff D., Smith C.C., Abate A.R. (2020). Joint Profiling of DNA and Proteins in Single Cells to Dissect Genotype-Phenotype Associations in Leukemia. Nat. Commun..

[20] Ni X., Zhuo M., Su Z., Duan J., Gao Y., Wang Z., Zong C., Bai H., Chapman A.R., Zhao J. (2013). Reproducible Copy Number Variation Patterns among Single Circulating Tumor Cells of Lung Cancer Patients. Proc. Natl. Acad. Sci. USA.

[21] Dago A.E., Stepansky A., Carlsson A., Luttgen M., Kendall J., Baslan T., Kolatkar A., Wigler M., Bethel K., Gross M.E. (2014). Rapid Phenotypic and Genomic Change in Response to Therapeutic Pressure in Prostate Cancer Inferred by High Content Analysis of Single Circulating Tumor Cells. PLoS ONE.

[22] Lohr J.G., Adalsteinsson V.A., Cibulskis K., Choudhury A.D., Rosenberg M., Cruz-Gordillo P., Francis J.M., Zhang C.-Z., Shalek A.K., Satija R. (2014). Whole-Exome Sequencing of Circulating Tumor Cells Provides a Window into Metastatic Prostate Cancer. Nat. Biotechnol..

[23] Lin D., Shen L., Luo M., Zhang K., Li J., Yang Q., Zhu F., Zhou D., Zheng S., Chen Y. (2021). Circulating Tumor Cells: Biology and Clinical Significance. Signal Transduct. Target. Ther..

[24] Ju S., Chen C., Zhang J., Xu L., Zhang X., Li Z., Chen Y., Zhou J., Ji F., Wang L. (2022). Detection of Circulating Tumor Cells: Opportunities and Challenges. Biomark. Res..

[25] Polzer B., Medoro G., Pasch S., Fontana F., Zorzino L., Pestka A., Andergassen U., Meier-Stiegen F., Czyz Z.T., Alberter B. (2014). Molecular Profiling of Single Circulating Tumor Cells with Diagnostic Intention. EMBO Mol. Med..

[26] Saadatpour A., Lai S., Guo G., Yuan G.-C. (2015). Single-Cell Analysis in Cancer Genomics. Trends Genet..

[27] Kharchenko P.V., Silberstein L., Scadden D.T. (2014). Bayesian Approach to Single-Cell Differential Expression Analysis. Nat. Methods.

[28] Shekhar K., Brodin P., Davis M.M., Chakraborty A.K. (2014). Automatic Classification of Cellular Expression by Nonlinear Stochastic Embedding (ACCENSE). Proc. Natl. Acad. Sci. USA.

[29] Wang Y., Waters J., Leung M.L., Unruh A., Roh W., Shi X., Chen K., Scheet P., Vattathil S., Liang H. (2014). Clonal Evolution in Breast Cancer Revealed by Single Nucleus Genome Sequencing. Nature.

[30] Wills Q.F., Mead A.J. (2015). Application of Single-Cell Genomics in Cancer: Promise and Challenges. Hum. Mol. Genet..

[31] Chen S., Jiang W., Du Y., Yang M., Pan Y., Li H., Cui M. (2023). Single-Cell Analysis Technologies for Cancer Research: From Tumor-Specific Single Cell Discovery to Cancer Therapy. Front. Genet..

[32] Thiele J.-A., Pitule P., Hicks J., Kuhn P. (2019). Single-Cell Analysis of Circulating Tumor Cells. Methods Mol. Biol..

[33] Reza K.K., Dey S., Wuethrich A., Wang J., Behren A., Antaw F., Wang Y., Sina A.A.I., Trau M. (2021). In Situ Single Cell Proteomics Reveals Circulating Tumor Cell Heterogeneity during Treatment. ACS Nano.

[34] Chew V., Toh H.C., Abastado J.-P. (2012). Immune Microenvironment in Tumor Progression: Characteristics and Challenges for Therapy. J. Oncol..

[35] Bai R., Cui J. (2022). Development of Immunotherapy Strategies Targeting Tumor Microenvironment Is Fiercely Ongoing. Front. Immunol..

[36] Kim N., Eum H.H., Lee H.-O. (2021). Clinical Perspectives of Single-Cell RNA Sequencing. Biomolecules.

[37] Rajan S., Zaccaria S., Cannon M.V., Cam M., Gross A.C., Raphael B.J., Roberts R.D. (2023). Structurally Complex Osteosarcoma Genomes Exhibit Limited Heterogeneity within Individual Tumors and across Evolutionary Time. Cancer Res. Commun..

[38] Meyers S., Alberti-Servera L., Gielen O., Erard M., Swings T., De Bie J., Michaux L., Dewaele B., Boeckx N., Uyttebroeck A. (2022). Monitoring of Leukemia Clones in B-Cell Acute Lymphoblastic Leukemia at Diagnosis and During Treatment by Single-Cell DNA Amplicon Sequencing. Hemasphere.

[39] Xu L., Durruthy-Durruthy R., Eastburn D.J., Pellegrino M., Shah O., Meyer E., Zehnder J. (2019). Clonal Evolution and Changes in Two AML Patients Detected with A Novel Single-Cell DNA Sequencing Platform. Sci. Rep..

[40] Borgsmüller N., Bonet J., Marass F., Gonzalez-Perez A., Lopez-Bigas N., Beerenwinkel N. (2020). BnpC: Bayesian Non-Parametric Clustering of Single-Cell Mutation Profiles. Bioinformatics.

[41] Huang A.Y., Lee E.A. (2021). Identification of Somatic Mutations From Bulk and Single-Cell Sequencing Data. Front Aging.

[42] Tang J., Tu K., Lu K., Zhang J., Luo K., Jin H., Wang L., Yang L., Xiao W., Zhang Q. (2021). Single-Cell Exome Sequencing Reveals Multiple Subclones in Metastatic Colorectal Carcinoma. Genome Med..

[43] Jaberi E., Tresse E., Grønbæk K., Weischenfeldt J., Issazadeh-Navikas S. (2020). Identification of Unique and Shared Mitochondrial DNA Mutations in Neurodegeneration and Cancer by Single-Cell Mitochondrial DNA Structural Variation Sequencing (MitoSV-Seq). EBioMedicine.

[44] Gráf A., Enyedi M.Z., Pintér L., Kriston-Pál É., Jaksa G., Bálind Á., Ezer É., Horváth P., Sükösd F., Kiss E. (2021). The Combination of Single-Cell and Next-Generation Sequencing Can Reveal Mosaicism for BRCA2 Mutations and the Fine Molecular Details of Tumorigenesis. Cancers.

[45] Liu L., Zhang Q., Wang C., Guo H., Mukwaya V., Chen R., Xu Y., Wei X., Chen X., Zhang S. (2023). Single-Cell Diagnosis of Cancer Drug Resistance through the Differential Endocytosis of Nanoparticles between Drug-Resistant and Drug-Sensitive Cancer Cells. ACS Nano.

[46] Pang L., Ding J., Ge Y., Fan J., Fan S.-K. (2019). Single-Cell-Derived Tumor-Sphere Formation and Drug-Resistance Assay Using an Integrated Microfluidics. Anal. Chem..

[47] Lee P., Yim R., Fung S.-H., Miu K.-K., Wang Z., Wu K.-C., Au L., Leung G.M.-K., Lee V.H.-F., Gill H. (2022). Single-Nucleotide Variations, Insertions/Deletions and Copy Number Variations in Myelodysplastic Syndrome during Disease Progression Revealed by a Single-Cell DNA Sequencing Platform. Int. J. Mol. Sci..

[48] Peretz C.A.C., McGary L.H.F., Kumar T., Jackson H., Jacob J., Durruthy-Durruthy R., Levis M.J., Perl A., Huang B.J., Smith C.C. (2021). Single-Cell DNA Sequencing Reveals Complex Mechanisms of Resistance to Quizartinib. Blood Adv..

[49] Grant C.R., Benjamin D.J., Cramer S., Rezazadeh Kalebasty A. (2024). A Remnant Never Forgotten: The Utility of Circulating Tumor DNA in Treatment Guidance of Urachal Cancer. Ther. Adv. Med. Oncol..

[50] Shen X., Dai J., Guo L., Liu Z., Yang L., Gu D., Xie Y., Wang Z., Li Z., Xu H. (2024). Single-Cell Low-Pass Whole Genome Sequencing Accurately Detects Circulating Tumor Cells for Liquid Biopsy-Based Multi-Cancer Diagnosis. NPJ Precis Oncol..

[51] Wang Z., Zhao Y., Shen X., Zhao Y., Zhang Z., Yin H., Zhao X., Liu H., Shi Q. (2022). Single-Cell Genomics-Based Molecular Algorithm for Early Cancer Detection. Anal. Chem..

[52] Liu Y., Luo G., Yan Y., Peng J. (2022). A Pan-Cancer Analysis of Copper Homeostasis-Related Gene Lipoyltransferase 1: Its Potential Biological Functions and Prognosis Values. Front. Genet..

[53] Zhang K., Chen Y., Zhu J., Ge X., Wu J., Xu P., Yao J. (2023). Advancement of Single-Cell Sequencing for Clinical Diagnosis and Treatment of Pancreatic Cancer. Front. Med..

[54] Tang X., Zhang Y., Zhang H., Zhang N., Dai Z., Cheng Q., Li Y. (2024). Single-Cell Sequencing: High-Resolution Analysis of Cellular Heterogeneity in Autoimmune Diseases. Clin. Rev. Allergy Immunol..

[55] Yuan P., Yan L.-Y., Qiao J. (2017). Application of Single-Cell Sequencing Technologies in Reproductive Medicine. Reprod. Dev. Med..

[56] Yadav S., Mehta P., Soni J., Chattopadhyay P., Devi P., Habyarimana T., Tardalkar K., Joshi M., Pandey R. (2023). Single-Cell RNA-Seq Reveals Intracellular Microbial Diversity within Immune Cells during SARS-CoV-2 Infection and Recovery. iScience.

[57] Kuksin M., Morel D., Aglave M., Danlos F.-X., Marabelle A., Zinovyev A., Gautheret D., Verlingue L. (2021). Applications of Single-Cell and Bulk RNA Sequencing in Onco-Immunology. Eur. J. Cancer.

[58] Lähnemann D., Köster J., Szczurek E., McCarthy D.J., Hicks S.C., Robinson M.D., Vallejos C. (2020). Eleven Grand Challenges in Single-Cell Data Science. Genome Biol..

[59] Tang F., Barbacioru C., Wang Y., Nordman E., Lee C., Xu N., Wang X., Bodeau J., Tuch B.B., Siddiqui A. (2009). mRNA-Seq Whole-Transcriptome Analysis of a Single Cell. Nat. Methods.

[60] Ren X., Zhou C., Lu Y., Ma F., Fan Y., Wang C. (2021). Single-Cell RNA-Seq Reveals Invasive Trajectory and Determines Cancer Stem Cell-Related Prognostic Genes in Pancreatic Cancer. Bioengineered.

[61] Boesch M., Sopper S., Zeimet A.G., Reimer D., Gastl G., Ludewig B., Wolf D. (2016). Heterogeneity of Cancer Stem Cells: Rationale for Targeting the Stem Cell Niche. Biochim. Biophys. Acta.

[62] Pan X.-W., Zhang H., Xu D., Chen J.-X., Chen W.-J., Gan S.-S., Qu F.-J., Chu C.-M., Cao J.-W., Fan Y.-H. (2020). Identification of a Novel Cancer Stem Cell Subpopulation That Promotes Progression of Human Fatal Renal Cell Carcinoma by Single-Cell RNA-Seq Analysis. Int. J. Biol. Sci..

[63] Segerstolpe Å., Palasantza A., Eliasson P., Andersson E.-M., Andréasson A.-C., Sun X., Picelli S., Sabirsh A., Clausen M., Bjursell M.K. (2016). Single-Cell Transcriptome Profiling of Human Pancreatic Islets in Health and Type 2 Diabetes. Cell Metab..

[64] Rosas P.C., Nagaraja G.M., Kaur P., Panossian A., Wickman G., Garcia L.R., Al-Khamis F.A., Asea A.A.A. (2016). Hsp72 (HSPA1A) Prevents Human Islet Amyloid Polypeptide Aggregation and Toxicity: A New Approach for Type 2 Diabetes Treatment. PLoS ONE.

[65] El-Sayes N., Vito A., Mossman K. (2021). Tumor Heterogeneity: A Great Barrier in the Age of Cancer Immunotherapy. Cancers.

[66] Lenz G., Onzi G.R., Lenz L.S., Buss J.H., Dos Santos J.A., Begnini K.R. (2022). The Origins of Phenotypic Heterogeneity in Cancer. Cancer Res..

[67] Wen L., Li G., Huang T., Geng W., Pei H., Yang J., Zhu M., Zhang P., Hou R., Tian G. (2022). Single-Cell Technologies: From Research to Application. Innovation (Camb).

[68] Chen H., Ye F., Guo G. (2019). Revolutionizing Immunology with Single-Cell RNA Sequencing. Cell. Mol. Immunol..

[69] Zhang Y., Wang D., Peng M., Tang L., Ouyang J., Xiong F., Guo C., Tang Y., Zhou Y., Liao Q. (2021). Single-cell RNA Sequencing in Cancer Research. J. Exp. Clin. Cancer Res..

[70] Wouters J., Kalender-Atak Z., Minnoye L., Spanier K.I., De Waegeneer M., Bravo González-Blas C., Mauduit D., Davie K., Hulselmans G., Najem A. (2020). Robust Gene Expression Programs Underlie Recurrent Cell States and Phenotype Switching in Melanoma. Nat. Cell Biol..

[71] Schreiber R.D., Old L.J., Smyth M.J. (2011). Cancer Immunoediting: Integrating Immunity’s Roles in Cancer Suppression and Promotion. Science.

[72] Hui Z., Ren Y., Zhang D., Chen Y., Yu W., Cao J., Liu L., Wang T., Xiao S., Zheng L. (2023). PD-1 Blockade Potentiates Neoadjuvant Chemotherapy in NSCLC via Increasing CD127+ and KLRG1+ CD8 T Cells. NPJ Precis. Oncol..

[73] Rajewsky N., Almouzni G., Gorski S.A., Aerts S., Amit I., Bertero M.G., Bock C., Bredenoord A.L., Cavalli G., Chiocca S. (2020). LifeTime and Improving European Healthcare through Cell-Based Interceptive Medicine. Nature.

[74] Stein C.M., Weiskirchen R., Damm F., Strzelecka P.M. (2021). Single-Cell Omics: Overview, Analysis, and Application in Biomedical Science. J. Cell. Biochem..

[75] Sade-Feldman M., Yizhak K., Bjorgaard S.L., Ray J.P., de Boer C.G., Jenkins R.W., Lieb D.J., Chen J.H., Frederick D.T., Barzily-Rokni M. (2018). Defining T Cell States Associated with Response to Checkpoint Immunotherapy in Melanoma. Cell.

[76] Van de Sande B., Lee J.S., Mutasa-Gottgens E., Naughton B., Bacon W., Manning J., Wang Y., Pollard J., Mendez M., Hill J. (2023). Applications of Single-Cell RNA Sequencing in Drug Discovery and Development. Nat. Rev. Drug Discov..

[77] Jang J.S., Li Y., Mitra A.K., Bi L., Abyzov A., van Wijnen A.J., Baughn L.B., Van Ness B., Rajkumar V., Kumar S. (2019). Molecular Signatures of Multiple Myeloma Progression through Single Cell RNA-Seq. Blood Cancer J..

[78] Tanaka N., Katayama S., Reddy A., Nishimura K., Niwa N., Hongo H., Ogihara K., Kosaka T., Mizuno R., Kikuchi E. (2018). Single-Cell RNA-Seq Analysis Reveals the Platinum Resistance Gene COX7B and the Surrogate Marker CD63. Cancer Med..

[79] Jerby-Arnon L., Shah P., Cuoco M.S., Rodman C., Su M.-J., Melms J.C., Leeson R., Kanodia A., Mei S., Lin J.-R. (2018). A Cancer Cell Program Promotes T Cell Exclusion and Resistance to Checkpoint Blockade. Cell.

[80] Cohen Y.C., Zada M., Wang S.-Y., Bornstein C., David E., Moshe A., Li B., Shlomi-Loubaton S., Gatt M.E., Gur C. (2021). Identification of Resistance Pathways and Therapeutic Targets in Relapsed Multiple Myeloma Patients through Single-Cell Sequencing. Nat. Med..

[81] Vandereyken K., Sifrim A., Thienpont B., Voet T. (2023). Methods and Applications for Single-Cell and Spatial Multi-Omics. Nat. Rev. Genet..

[82] Bekaert B., Boel A., Cosemans G., De Witte L., Menten B., Heindryckx B. (2022). CRISPR/Cas Gene Editing in the Human Germline. Semin. Cell Dev. Biol..

[83] Vishnubalaji R., Alajez N.M. (2023). Single-Cell Transcriptome Analysis Revealed Heterogeneity and Identified Novel Therapeutic Targets for Breast Cancer Subtypes. Cells.

[84] Meyers R.M., Bryan J.G., McFarland J.M., Weir B.A., Sizemore A.E., Xu H., Dharia N.V., Montgomery P.G., Cowley G.S., Pantel S. (2017). Computational Correction of Copy Number Effect Improves Specificity of CRISPR-Cas9 Essentiality Screens in Cancer Cells. Nat. Genet..

[85] Rambow F., Rogiers A., Marin-Bejar O., Aibar S., Femel J., Dewaele M., Karras P., Brown D., Chang Y.H., Debiec-Rychter M. (2018). Toward Minimal Residual Disease-Directed Therapy in Melanoma. Cell.

[86] Petti A.A., Williams S.R., Miller C.A., Fiddes I.T., Srivatsan S.N., Chen D.Y., Fronick C.C., Fulton R.S., Church D.M., Ley T.J. (2019). A General Approach for Detecting Expressed Mutations in AML Cells Using Single Cell RNA-Sequencing. Nat. Commun..

[87] Xu X., Hou Y., Yin X., Bao L., Tang A., Song L., Li F., Tsang S., Wu K., Wu H. (2012). Single-Cell Exome Sequencing Reveals Single-Nucleotide Mutation Characteristics of a Kidney Tumor. Cell.

[88] Gao Y., Ni X., Guo H., Su Z., Ba Y., Tong Z., Guo Z., Yao X., Chen X., Yin J. (2017). Single-Cell Sequencing Deciphers a Convergent Evolution of Copy Number Alterations from Primary to Circulating Tumor Cells. Genome Res..

[89] Wu C.-Y., Lau B.T., Kim H.S., Sathe A., Grimes S.M., Ji H.P., Zhang N.R. (2021). Integrative Single-Cell Analysis of Allele-Specific Copy Number Alterations and Chromatin Accessibility in Cancer. Nat. Biotechnol..

[90] Aganezov S., Goodwin S., Sherman R.M., Sedlazeck F.J., Arun G., Bhatia S., Lee I., Kirsche M., Wappel R., Kramer M. (2020). Comprehensive Analysis of Structural Variants in Breast Cancer Genomes Using Single-Molecule Sequencing. Genome Res..

[91] Funnell T., O’Flanagan C.H., Williams M.J., McPherson A., McKinney S., Kabeer F., Lee H., Salehi S., Vázquez-García I., Shi H. (2022). Single-Cell Genomic Variation Induced by Mutational Processes in Cancer. Nature.

[92] Shema E., Bernstein B.E., Buenrostro J.D. (2019). Single-Cell and Single-Molecule Epigenomics to Uncover Genome Regulation at Unprecedented Resolution. Nat. Genet..

[93] Schuster L.C. (2022). Dissecting Heterogeneity, Clonal Evolution, and Epigenetic Changes in Distinct and Molecularly Defined AML Subsets by Multi Omics Single-Cell Sequencing. Heidelberg. https://archiv.ub.uni-heidelberg.de/.

[94] Misra P., Jadhav A.R., Bapat S.A. (2022). Single-Cell Sequencing: A Cutting Edge Tool in Molecular Medical Research. Armed Forces Med. J. India.

[95] Wang T., Dang N., Tang G., Li Z., Li X., Shi B., Xu Z., Li L., Yang X., Xu C. (2022). Integrating Bulk and Single-Cell RNA Sequencing Reveals Cellular Heterogeneity and Immune Infiltration in Hepatocellular Carcinoma. Mol. Oncol..

[96] Forcato M., Romano O., Bicciato S. (2021). Computational Methods for the Integrative Analysis of Single-Cell Data. Brief. Bioinform..

[97] Huang H., Wu F., Yu Y., Xu B., Chen D., Huo Y., Li S. (2024). Multi-Transcriptomics Analysis of Microvascular Invasion-Related Malignant Cells and Development of a Machine Learning-Based Prognostic Model in Hepatocellular Carcinoma. Front. Immunol..

[98] Kumar M. (2023). The Precision Oncology Approach to Molecular Cancer Therapeutics Targeting Oncogenic Signaling Pathways Is a Means to an End. arXiv.

[99] Sierant M.C., Choi J. (2018). Single-Cell Ssequencing in Cancer: Recent Applications to Immunogenomics and Multi-Omics Tools. Genomics Inform..

[100] Zhang A.W., Campbell K.R. (2020). Computational Modelling in Single-Cell Cancer Genomics: Methods and Future Directions. Phys. Biol..

[101] Zhang C., Lei L., Yang X., Ma K., Zheng H., Su Y., Jiao A., Wang X., Liu H., Zou Y. (2021). Single-Cell Sequencing Reveals Antitumor Characteristics of Intratumoral Immune Cells in Old Mice. J. Immunother. Cancer.

[102] Chen Y.-Z., Meng Z.-S., Xiang Z.-L. (2024). HMGB2 Drives Tumor Progression and Shapes the Immunosuppressive Microenvironment in Hepatocellular Carcinoma: Insights from Multi-Omics Analysis. Front. Immunol..

[103] Wolfe C., Feng Y., Chen D., Purcell E., Talkington A., Dolatshahi S., Shakeri H. GeoTyper: Automated Pipeline from Raw scRNA-Seq Data to Cell Type Identification. Proceedings of the 2022 Systems and Information Engineering Design Symposium (SIEDS).

[104] Wang S., Sun S.-T., Zhang X.-Y., Ding H.-R., Yuan Y., He J.-J., Wang M.-S., Yang B., Li Y.-B. (2023). The Evolution of Single-Cell RNA Sequencing Technology and Application: Progress and Perspectives. Int. J. Mol. Sci..

[105] Pires A., Greenshields-Watson A., Jones E., Smart K., Lauder S.N., Somerville M., Milutinovic S., Kendrick H., Hindley J.P., French R. (2020). Immune Remodeling of the Extracellular Matrix Drives Loss of Cancer Stem Cells and Tumor Rejection. Cancer Immunol. Res..

[106] Anderson N.M., Simon M.C. (2020). The Tumor Microenvironment. Curr. Biol..

[107] Alcantara M.B., Tang W.S., Wang D., Kaniowski D., Kang E., Dizman N., Chehrazi-Raffle A., Meza L., Zengin Z., Hall J. (2023). Targeting STAT3 in Tumor-Associated Antigen-Presenting Cells as a Strategy for Kidney and Bladder Cancer Immunotherapy. Front. Immunol..

[108] Li L., Yu R., Cai T., Chen Z., Lan M., Zou T., Wang B., Wang Q., Zhao Y., Cai Y. (2020). Effects of Immune Cells and Cytokines on Inflammation and Immunosuppression in the Tumor Microenvironment. Int. Immunopharmacol..

[109] Kumar V., Ramnarayanan K., Sundar R., Padmanabhan N., Srivastava S., Koiwa M., Yasuda T., Koh V., Huang K.K., Tay S.T. (2022). Single-Cell Atlas of Lineage States, Tumor Microenvironment, and Subtype-Specific Expression Programs in Gastric Cancer. Cancer Discov..

[110] Nofech-Mozes I., Soave D., Awadalla P., Abelson S. (2023). Pan-Cancer Classification of Single Cells in the Tumour Microenvironment. Nat. Commun..

[111] Lee J.J., Bernard V., Semaan A., Monberg M.E., Huang J., Stephens B.M., Lin D., Rajapakshe K.I., Weston B.R., Bhutani M.S. (2021). Elucidation of Tumor-Stromal Heterogeneity and the Ligand-Receptor Interactome by Single-Cell Transcriptomics in Real-World Pancreatic Cancer Biopsies. Clin. Cancer Res..

[112] Qian J., Olbrecht S., Boeckx B., Vos H., Laoui D., Etlioglu E., Wauters E., Pomella V., Verbandt S., Busschaert P. (2020). A Pan-Cancer Blueprint of the Heterogeneous Tumor Microenvironment Revealed by Single-Cell Profiling. Cell Res..

[113] Zhang J., Song C., Tian Y., Yang X. (2021). Single-Cell RNA Sequencing in Lung Cancer: Revealing Phenotype Shaping of Stromal Cells in the Microenvironment. Front. Immunol..

[114] Gao Y., Li H., Li Z., Xie L., Liu X., Huang Z., Chen B., Lin X., Wang X., Zheng Y. (2021). Single-Cell Analysis Reveals the Heterogeneity of Monocyte-Derived and Peripheral Type-2 Conventional Dendritic Cells. J. Immunol..

[115] Sun D., Guan X., Moran A.E., Wu L.-Y., Qian D.Z., Schedin P., Dai M.-S., Danilov A.V., Alumkal J.J., Adey A.C. (2022). Identifying Phenotype-Associated Subpopulations by Integrating Bulk and Single-Cell Sequencing Data. Nat. Biotechnol..

[116] Liu J., Xu T., Jin Y., Huang B., Zhang Y. (2020). Progress and Clinical Application of Single-Cell Transcriptional Sequencing Technology in Cancer Research. Front. Oncol..

[117] Ke M., Elshenawy B., Sheldon H., Arora A., Buffa F.M. (2022). Single Cell RNA-Sequencing: A Powerful yet Still Challenging Technology to Study Cellular Heterogeneity. Bioessays.

[118] Zappia L., Theis F.J. (2021). Over 1000 Tools Reveal Trends in the Single-Cell RNA-Seq Analysis Landscape. Genome Biol..

[119] Chen G., Ning B., Shi T. (2019). Single-Cell RNA-Seq Technologies and Related Computational Data Analysis. Front. Genet..

[120] Gross A., Schoendube J., Zimmermann S., Steeb M., Zengerle R., Koltay P. (2015). Technologies for Single-Cell Isolation. Int. J. Mol. Sci..

[121] Wolfien M., David R., Galow A.-M. (2021). Single-Cell RNA Sequencing Procedures and Data Analysis. Bioinformatics.

[122] Vieth B., Parekh S., Ziegenhain C., Enard W., Hellmann I. (2019). A Systematic Evaluation of Single Cell RNA-Seq Analysis Pipelines. Nat. Commun..

[123] Tjoonk N. What Is CellRanger and How Do You Use It?. https://www.scdiscoveries.com/blog/knowledge/cellranger/.

[124] Wolf F.A., Angerer P., Theis F.J. (2018). SCANPY: Large-Scale Single-Cell Gene Expression Data Analysis. Genome Biol..

[125] Hao Y., Stuart T., Kowalski M.H., Choudhary S., Hoffman P., Hartman A., Srivastava A., Molla G., Madad S., Fernandez-Granda C. (2024). Dictionary Learning for Integrative, Multimodal and Scalable Single-Cell Analysis. Nat. Biotechnol..

[126] Risso D., Purvis L., Fletcher R.B., Das D., Ngai J., Dudoit S., Purdom E. (2018). clusterExperiment and RSEC: A Bioconductor Package and Framework for Clustering of Single-Cell and Other Large Gene Expression Datasets. PLoS Comput. Biol..

[127] Amezquita R.A., Lun A.T.L., Becht E., Carey V.J., Carpp L.N., Geistlinger L., Marini F., Rue-Albrecht K., Risso D., Soneson C. (2020). Orchestrating Single-Cell Analysis with Bioconductor. Nat. Methods.

[128] Wickham H., Averick M., Bryan J., Chang W., McGowan L., François R., Grolemund G., Hayes A., Henry L., Hester J. (2019). Welcome to the Tidyverse. J. Open Source Softw..

[129] Kiselev V.Y., Yiu A., Hemberg M. (2017). Scmap—A Tool for Unsupervised Projection of Single Cell RNA-Seq Data. bioRxiv.

[130] Welch J.D., Kozareva V., Ferreira A., Vanderburg C., Martin C., Macosko E.Z. (2019). Single-Cell Multi-Omic Integration Compares and Contrasts Features of Brain Cell Identity. Cell.

[131] Lin Y., Ghazanfar S., Wang K.Y.X., Gagnon-Bartsch J.A., Lo K.K., Su X., Han Z.-G., Ormerod J.T., Speed T.P., Yang P. (2019). scMerge Leverages Factor Analysis, Stable Expression, and Pseudoreplication to Merge Multiple Single-Cell RNA-Seq Datasets. Proc. Natl. Acad. Sci. USA.

[132] Wang C., Sun D., Huang X., Wan C., Li Z., Han Y., Qin Q., Fan J., Qiu X., Xie Y. (2020). Integrative Analyses of Single-Cell Transcriptome and Regulome Using MAESTRO. Genome Biol..

[133] Lun A., Griffiths J., McCarthy D. DropletUtils: Utilities for Handling Single-Cell Droplet Data; Bioconductor Version 3.13; 2021.

[134] Zappia L L.A. (2024). zellkonverter: Conversion Between scRNA-Seq Objects.

[135] Yip S.H., Wang P., Kocher J.-P.A., Sham P.C., Wang J. (2017). Linnorm: Improved Statistical Analysis for Single Cell RNA-Seq Expression Data. Nucleic Acids Res..

[136] Ruan X., Lai C., Li L., Wang B., Lu X., Zhang D., Fang J., Lai M., Yan F. (2024). Integrative Analysis of Single-Cell and Bulk Multi-Omics Data to Reveal Subtype-Specific Characteristics and Therapeutic Strategies in Clear Cell Renal Cell Carcinoma Patients. J. Cancer.

[137] Xing Z., Lin D., Hong Y., Ma Z., Jiang H., Lu Y., Sun J., Song J., Xie L., Yang M. (2023). Construction of a Prognostic 6-Gene Signature for Breast Cancer Based on Multi-Omics and Single-Cell Data. Front. Oncol..

[138] Warfvinge R., Geironson Ulfsson L., Dhapola P., Safi F., Sommarin M.N.E., Soneji S., Hjorth-Hansen H., Mustjoki S., Richter J., Krishna Thakur R. (2023). Single Cell Multi-Omics Analysis of Chronic Myeloid Leukemia Links Cellular Heterogeneity to Therapy Response. bioRxiv.

[139] Argelaguet R., Arnol D., Bredikhin D., Deloro Y., Velten B., Marioni J.C., Stegle O. (2020). MOFA+: A Statistical Framework for Comprehensive Integration of Multi-Modal Single-Cell Data. Genome Biol..

[140] Lotfollahi M., Naghipourfar M., Luecken M.D., Khajavi M., Büttner M., Wagenstetter M., Avsec Ž., Gayoso A., Yosef N., Interlandi M. (2022). Mapping Single-Cell Data to Reference Atlases by Transfer Learning. Nat. Biotechnol..

[141] Zhang J., Zhang L., Gongol B., Hayes J., Borowsky A.T., Bailey-Serres J., Girke T. (2024). spatialHeatmap: Visualizing Spatial Bulk and Single-Cell Assays in Anatomical Images. NAR Genom. Bioinform..

[142] Canete N.P., Iyengar S.S., Wilmott J.S., Ormerod J.T., Harman A.N., Patrick E. (2021). spicyR: Spatial Analysis of *in Situ* Cytometry Data in R. bioRxiv.

[143] Righelli D., Weber L.M., Crowell H.L., Pardo B., Collado-Torres L., Ghazanfar S., Lun A.T.L., Hicks S.C., Risso D. (2022). SpatialExperiment: Infrastructure for Spatially-Resolved Transcriptomics Data in R Using Bioconductor. Bioinformatics.

[144] Lun A. (2024). bluster: Clustering Algorithms for Bioconductor.

[145] Campbell J., Corbett S., Koga Y., Yang S., Reed E., Wang Z. (2021). Celda: CEllular Latent Dirichlet Allocation, R package version.

[146] Kiselev V.Y., Kirschner K., Schaub M.T., Andrews T., Yiu A., Chandra T., Natarajan K.N., Reik W., Barahona M., Green A.R. (2017). SC3: Consensus Clustering of Single-Cell RNA-Seq Data. Nat. Methods.

[147] Miao Z., Deng K., Wang X., Zhang X. (2018). DEsingle for Detecting Three Types of Differential Expression in Single-Cell RNA-Seq Data. Bioinformatics.

[148] Tiberi S., Crowell H.L., Samartsidis P., Weber L.M., Robinson M.D. (2020). *distinct*: A Novel Approach to Differential Distribution Analyses. bioRxiv.

[149] Traag V.A., Waltman L., van Eck N.J. (2019). From Louvain to Leiden: Guaranteeing Well-Connected Communities. Sci. Rep..

[150] Aibar S., González-Blas C.B., Moerman T., Huynh-Thu V.A., Imrichova H., Hulselmans G., Rambow F., Marine J.-C., Geurts P., Aerts J. (2017). SCENIC: Single-Cell Regulatory Network Inference and Clustering. Nat. Methods.

[151] Gao R., Bai S., Henderson Y.C., Lin Y., Schalck A., Yan Y., Kumar T., Hu M., Sei E., Davis A. (2021). Delineating Copy Number and Clonal Substructure in Human Tumors from Single-Cell Transcriptomes. Nat. Biotechnol..

[152] Khayatan D., Hussain A., Tebyaniyan H. (2023). Exploring Animal Models in Oral Cancer Research and Clinical Intervention: A Critical Review. Vet. Med. Sci..

[153] Miao Y., Wang P., Huang J., Qi X., Liang Y., Zhao W., Wang H., Lyu J., Zhu H. (2024). Metabolomics, Transcriptome and Single-Cell RNA Sequencing Analysis of the Metabolic Heterogeneity between Oral Cancer Stem Cells and Differentiated Cancer Cells. Cancers.

[154] Aran D., Looney A.P., Liu L., Wu E., Fong V., Hsu A., Chak S., Naikawadi R.P., Wolters P.J., Abate A.R. (2019). Reference-Based Analysis of Lung Single-Cell Sequencing Reveals a Transitional Profibrotic Macrophage. Nat. Immunol..

[155] Garvin T., Aboukhalil R., Kendall J., Baslan T., Atwal G.S., Hicks J., Wigler M., Schatz M.C. (2015). Interactive Analysis and Assessment of Single-Cell Copy-Number Variations. Nat. Methods.

[156] Zaccaria S., Raphael B.J. (2021). Characterizing Allele- and Haplotype-Specific Copy Numbers in Single Cells with CHISEL. Nat. Biotechnol..

[157] Roth A., Khattra J., Yap D., Wan A., Laks E., Biele J., Ha G., Aparicio S., Bouchard-Côté A., Shah S.P. (2014). PyClone: Statistical Inference of Clonal Population Structure in Cancer. Nat. Methods.

[158] Dong X., Zhang L., Hao X., Wang T., Vijg J. (2020). SCCNV: A Software Tool for Identifying Copy Number Variation from Single-Cell Whole-Genome Sequencing. Front. Genet..

[159] Dong X., Zhang L., Milholland B., Lee M., Maslov A.Y., Wang T., Vijg J. (2017). Accurate Identification of Single-Nucleotide Variants in Whole-Genome-Amplified Single Cells. Nat. Methods.

[160] Jahn K., Kuipers J., Beerenwinkel N. (2016). Tree Inference for Single-Cell Data. Genome Biol..

[161] Erfanian N., Heydari A.A., Feriz A.M., Iañez P., Derakhshani A., Ghasemigol M., Farahpour M., Razavi S.M., Nasseri S., Safarpour H. (2023). Deep Learning Applications in Single-Cell Genomics and Transcriptomics Data Analysis. Biomed. Pharmacother..

[162] Molho D., Ding J., Tang W., Li Z., Wen H., Wang Y., Venegas J., Jin W., Liu R., Su R. (2024). Deep Learning in Single-Cell Analysis. ACM Trans. Intell. Syst. Technol..

[163] Premkumar R., Srinivasan A., Harini Devi K.G., M D., E G., Jadhav P., Futane A., Narayanamurthy V. (2024). Single-Cell Classification, Analysis, and Its Application Using Deep Learning Techniques. Biosystems.

[164] Zhu Y., Bai L., Ning Z., Fu W., Liu J., Jiang L., Fei S., Gong S., Lu L., Deng M. (2024). Deep Learning for Clustering Single-Cell RNA-Seq Data. Curr. Bioinform..

[165] Halawani R., Buchert M., Chen Y.-P.P. (2023). Deep Learning Exploration of Single-Cell and Spatially Resolved Cancer Transcriptomics to Unravel Tumour Heterogeneity. Comput. Biol. Med..

[166] Qi R., Zou Q. (2023). Trends and Potential of Machine Learning and Deep Learning in Drug Study at Single-Cell Level. Research.

[167] Liu J., Zhang P., Yang F., Jiang K., Sun S., Xia Z., Yao G., Tang J. (2023). Integrating Single-Cell Analysis and Machine Learning to Create Glycosylation-Based Gene Signature for Prognostic Prediction of Uveal Melanoma. Front. Endocrinol. (Lausanne).

[168] Mou L., Pu Z., Luo Y., Quan R., So Y., Jiang H. (2023). Construction of a Lipid Metabolism-Related Risk Model for Hepatocellular Carcinoma by Single Cell and Machine Learning Analysis. Front. Immunol..

[169] Chen H., Yang W., Ma L., Li Y., Ji Z. (2023). Machine-Learning Based Integrating Bulk and Single-Cell RNA Sequencing Reveals the SLC38A5-CCL5 Signaling as a Promising Target for Clear Cell Renal Cell Carcinoma Treatment. Transl. Oncol..

[170] Kang Z., Zhao Y.-X., Qiu R.S.Q., Chen D.-N., Zheng Q.-S., Xue X.-Y., Xu N., Wei Y. (2024). Identification Macrophage Signatures in Prostate Cancer by Single-Cell Sequencing and Machine Learning. Cancer Immunol. Immunother..

[171] He X., Liu X., Zuo F., Shi H., Jing J. (2023). Artificial Intelligence-Based Multi-Omics Analysis Fuels Cancer Precision Medicine. Semin. Cancer Biol..

[172] Li J., Li L., You P., Wei Y., Xu B. (2023). Towards Artificial Intelligence to Multi-Omics Characterization of Tumor Heterogeneity in Esophageal Cancer. Semin. Cancer Biol..

[173] Danishuddin, Khan S., Kim J.J. (2024). From Cancer Big Data to Treatment: Artificial Intelligence in Cancer Research. J. Gene Med..

[174] Luo J., Pan M., Mo K., Mao Y., Zou D. (2023). Emerging Role of Artificial Intelligence in Diagnosis, Classification and Clinical Management of Glioma. Semin. Cancer Biol..

